# Most of the long-term genetic gain from optimum-contribution selection can be realised with restrictions imposed during optimisation

**DOI:** 10.1186/s12711-015-0107-7

**Published:** 2015-03-28

**Authors:** Mark Henryon, Tage Ostersen, Birgitte Ask, Anders C Sørensen, Peer Berg

**Affiliations:** Seges, Danish Pig Research Centre, Axeltorv 3, 1609 Copenhagen V, Denmark; School of Animal Biology, University of Western Australia, 35 Stirling Highway, Crawley, WA 6009 Australia; Aarhus University, Institute for Molecular Biology and Genetics, P.O. Box 50, 8830 Tjele, Denmark; NordGen, Nordic Genetic Resource Center, P.O. Box 115, 1431 Ås, Norway

## Abstract

**Background:**

We tested the hypothesis that optimum-contribution selection (OCS) with restrictions imposed during optimisation realises most of the long-term genetic gain realised by OCS without restrictions.

**Methods:**

We used stochastic simulation to estimate long-term rates of genetic gain realised by breeding schemes that applied OCS without and with restrictions imposed during optimisation, where long-term refers to generations 23 to 25 (approximately). Six restrictions were imposed. Five of these removed solutions from the solution space. The sixth removed records of selection decisions made at earlier selection times. We also simulated a conventional breeding scheme with truncation selection as a reference point. Generations overlapped, selection was for a single trait, and the trait was observed for all selection candidates prior to selection.

**Results:**

OCS with restrictions realised 67 to 99% of the additional gain realised by OCS without restrictions, where additional gain was the difference in the long-term rates of genetic gain realised by OCS without restrictions and our reference point with truncation selection. The only exceptions were those restrictions that removed all solutions near the optimum solution from the solution space and the restriction that removed records of selection decisions made at earlier selection times. Imposing these restrictions realised only −12 to 46% of the additional gain.

**Conclusions:**

Most of the long-term genetic gain realised by OCS without restrictions can be realised by OCS with restrictions imposed during optimisation, provided the restrictions do not remove all solutions near the optimum from the solution space and do not remove records of earlier selection decisions. In breeding schemes where OCS cannot be applied optimally because of biological and logistical restrictions, OCS with restrictions provides a useful alternative. Not only does it realise most of the long-term genetic gain, OCS with restrictions enables OCS to be tailored to individual breeding schemes.

## Background

Optimum-contribution selection (OCS) maximises the genetic merit of a cohort of animals while constraining the average relationship of the current generation [1-5]. OCS does this by optimising the genetic contribution (i.e., number of matings) of each selection candidate to the cohort, conditional on predicted breeding values and additive-genetic relationships. The benefit of OCS, besides reducing the risks of inbreeding, genetic drift, and undesirable changes in gene frequencies, is that it can maximise long-term genetic gain, which is the goal of most breeding schemes [2,6]. Maximising long-term genetic gain is realised by striking a balance between short-term rates of genetic gain and inbreeding. This promotes short-term genetic gain at rates of inbreeding that do not substantially erode additive-genetic variation [7]. Despite the benefit, OCS is not widely used in practical breeding schemes. As far as we are aware, it has only been applied by a few actors in progressive sectors of the breeding industry [8]. A major reason is that the optimum number of matings, as defined by OCS, cannot be allocated to all of the selected animals because of biological and logistical restrictions. In pig-breeding schemes, for example, it may only be possible to allocate optimum numbers of matings to selected sires because it is difficult to foresee which females will be available for reproduction at each selection time. Likewise, many pig breeders will only use sires with the highest breeding values for breeding. One way to make OCS more practical is to impose restrictions on OCS during optimisation. Not only would this make OCS decisions readily applicable to individual breeding schemes, it may even realise most, if not all, of the long-term genetic gain realised by OCS without restrictions for two reasons. First, in most OCS analyses, there are many ways to allocate numbers of matings to the selection candidates (i.e., many possible solutions in the solution space) [8,9]. It is likely that many solutions lie at, or near, the optimum solution. Imposing restrictions on OCS merely removes some of the solutions from the solution space, so solutions can still exist near the optimum. Second, OCS is able to correct for selection decisions made at earlier selection times by taking into account that some selection candidates and ancestral animals have already generated offspring [3,5]. Based on this line of reasoning, we hypothesised that OCS with restrictions imposed during optimisation will realise most of the long-term genetic gain realised by OCS without restrictions. We tested this hypothesis by stochastic simulation. We simulated breeding schemes with restrictions that were inspired by, but not unique, to pig breeding.

## Methods

### Procedure

We used stochastic simulation to estimate long-term rates of genetic gain realised by OCS without and with restrictions imposed during optimisation, where long-term refers to generations 23 to 25 (approximately). We did this by simulating breeding schemes that loosely resembled those used for pigs. Generations overlapped, selection was for a single trait, and the trait was observed for all selection candidates prior to selection. We also simulated a conventional breeding scheme with truncation selection as a reference point. In all schemes, best linear unbiased prediction (BLUP) breeding values were used as indicators of genetic merit. The breeding scheme that applied OCS without restrictions was *unrestricted OCS*.

#### Unrestricted OCS.

A total of 300 matings were allocated among approximately 2250 male and 2250 female selection candidates by OCS at time *t* to generate a new cohort of animals, where the time interval *t* to *t* + 1 represents a female reproductive cycle. Males were candidates for selection at ages 3 to 5 (i.e., born at times *t*-3 to *t*-5). There was no upper limit for the number of matings that were allocated to each male; males were allocated 0, 1, 2 … or 300 matings at each time. Females were candidates at ages 4 to 6 (i.e., born at times *t*-4 to *t*-6). Each female was allocated 0 or 1 mating at each time and 300 females were allocated a mating at each time.

OCS was carried out by maximising the genetic merit of the new cohort while applying a penalty to the average relationship of the current generation, which included the new cohort. Nine penalties were applied: 5, 10, 20, 50, 100, 200, 500, 1000, and 5000. The average relationship was calculated using an additive-relationship matrix that included male and female selection candidates, immature offspring that were too young to be candidates, and all ancestors traced back from these animals.

The 300 sire and dam matings were paired randomly. Each pairing (dam) produced five offspring, resulting in 300 full-sib families and 1500 offspring. Offspring were assigned as males and females with a probability of 0.5. All animals were phenotyped for the trait under selection at age 1 (i.e., born at time *t*-1).

The breeding schemes that applied OCS with restrictions were simulated by imposing restrictions on the breeding scheme, *unrestricted OCS*. An overview of these breeding schemes is in Table 1. Each breeding scheme was simulated at the nine penalties. The schemes with restrictions can be loosely grouped into three categories. In the first category, restrictions were imposed on female candidates. These schemes are *truncated dams* and *dams unknown*. In the second category, restrictions were imposed on both male and female candidates. These schemes are *one*-*chance OCS of sires*, *pre*-*selection of sires*, and *sire multiples*. In the third category, which only includes the scheme *offspring unknown*, immature offspring that were too young to be selection candidates were not known before OCS. The first and second categories are restrictions that remove solutions from the solution space. *Offspring unknown*, in the third category, removes records of selection decisions made at earlier times. We also simulated a breeding scheme, *multiple restrictions*, in which several of the restrictions that remove solutions from the solution space were imposed simultaneously. The following sections provide a description of the breeding schemes with restrictions.

**Table 1:** **Breeding schemes applying optimum**-**contribution selection** (**OCS**) **with restrictions**

	**Restrictions**					
**Breeding scheme**	**Truncated dams**	**Dams unknown**	**One**-**chance males**	**Pre**-**selection males**	**Sire multiples**	**Offspring unknown**
*Truncated dams*	●					
*Dams unknown*	●	●				
*One*-*chance OCS of sires*	●		●			
*Pre*-*selection of sires* 25%	●			25		
*Pre*-*selection of sires* 10%	●			10		
*Pre*-*selection of sires* 5%	●			5		
*Pre*-*selection of sires* 1%	●			1		
*Pre*-*selection of sires* 0.5%	●			0.5		
*Sire multiples* 5	●				5	
*Sire multiples* 10	●				10	
*Sire multiples* 20	●				20	
*Sire multiples* 50	●				50	
*Sire multiples* 100	●				100	
*Offspring unknown*	●					●
*Multiple restrictions*	●	●	●	5	10	

The restrictions are truncation selection of dams (Truncated dams), truncation-selected dams not known before carrying out OCS of sires (Dams unknown), OCS limited to young males (One-chance males), pre-selection of males by truncation selection before OCS (Pre-selection males), numbers of matings allocated to sires by OCS in multiples (Sire multiples), and immature offspring not known before OCS (Offspring unknown). Filled circles (●) and numbers indicate that restriction was imposed, where numbers indicate the proportion (%) of males that were pre-selected and the multiple used to allocate numbers of matings to sires.

#### Truncated dams.

*Truncated dams* is as for *unrestricted OCS* with the restriction that OCS was only applied to male candidates. Three-hundred dams were truncation selected based on breeding value at each time and each selected dam was allocated one mating. Sires were selected by OCS conditional on the truncation-selected dams. The selected dams were used to estimate the genetic merit of the new cohort and they were included in the additive-relationship matrix. The additive-relationship matrix included male candidates, truncation-selected dams, immature offspring, and all ancestral animals traced back from these animals.

#### Dams unknown.

*Dams unknown* is as for *truncated dams* with the added restriction that the truncation-selected dams were not known before carrying out OCS of sires. The truncation-selected dams were not used to estimate the genetic merit of the new cohort and they were excluded from the additive-relationship matrix. This breeding scheme resembles practical breeding schemes where it is difficult to foresee which females will be available for reproduction at each selection time.

#### One-chance OCS of sires.

*One*-*chance OCS of sires* is as for *truncated dams* with the added restriction that males were only candidates for OCS at age 3 (i.e., born at time *t*-3). Older males were not candidates for OCS. This breeding scheme resembles practical breeding schemes where males are only candidates for selection during the initial stages of their reproductive lives.

#### Pre-selection of sires.

*Pre*-*selection of sires* is as for *truncated dams* with the added restriction that 0.5, 1, 5, 10, and 25% of the male candidates were pre-selected by truncation selection based on breeding value before OCS. Only males that were pre-selected were candidates for OCS. This breeding scheme resembles practical breeding schemes where animal breeders will only use sires with the highest breeding values for breeding and/or it is expensive to maintain breeding animals.

#### Sire multiples.

*Sire multiples* is as for *truncated dams* with the added restriction that numbers of matings were allocated to sires in multiples of 5, 10, 20, 50, and 100. When the multiple was 5, for example, sires could only be allocated 0, 5, 10, … 300 matings. This breeding scheme resembles practical breeding schemes where the nucleus population is maintained in multiple herds and the number of matings allocated to each selected sire are distributed equally across herds.

#### Offspring unknown.

*Offspring unknown* is as for *truncated dams* with the added restriction that immature offspring, too young to be selection candidates, were not known before carrying out OCS of sires. The offspring were excluded from the additive-relationship matrix. The additive-relationship matrix only included male candidates, truncation-selected dams, and ancestral animals traced back from these animals. This restriction removes records of selection decisions made before time *t*. The breeding scheme resembles practical breeding schemes where young animals are first recorded in databases later in life.

#### Multiple restrictions.

Several of the restrictions that remove solutions from the solution space were imposed simultaneously:

- *Truncated dams*: OCS was only applied to male candidates and 300 dams were truncation selected at each time.
- *Dams unknown*: Truncation-selected dams were not known before carrying out OCS of sires.
- *One*-*chance OCS of sires*: Males were only candidates for OCS at age 3.
- *Pre*-*selection of sires 5%*: 5% of the male candidates were pre-selected before OCS.
- *Sire multiples* 10: Numbers of matings were allocated to sires in multiples of 10.

#### Truncation selection 10.

Truncation selection 10 is a conventional breeding scheme with 10 sires and 300 dams truncation selected based on breeding value at each time. Each sire was randomly mated with 30 dams and each dam (mating) produced five offspring that resulted in 300 full-sib families and 1500 offspring.

Breeding schemes were run for 100 times (*t* = 1, … 100, approximately 25 generations). In the first 20 times, truncation selection was carried out by applying *truncation selection* 10. This established a selected population before OCS was applied at times 21 to 100. We simulated 100 replicates for each breeding scheme. The breeding schemes that applied OCS were compared at penalties that maximised long-term rates of genetic gain for the trait under selection.

### Trait

The trait under selection was assumed to be normally-distributed and genetically controlled by the infinitesimal model of additive-genetic effects. It had a heritability of 0.20 and additive-genetic variance of 1.0.

### Sampling

Breeding schemes were initiated by sampling an unrelated base population of 20 sires and 600 dams. The phenotype of the trait for the *i*^th^ base animal, p_*i*_, was calculated as p_*i*_ = a_*i*_ + e_*i*_, where a_*i*_ is the base animal’s true additive-genetic value and e_*i*_ is its residual environmental value. The true additive-genetic value was sampled from ${\mathrm{a}}_i\sim N\left(0,{\sigma}_a^2 = 1\right)$ and the environmental value from ${\mathrm{e}}_i\sim N\left(0,{\displaystyle {\sigma}_e^2} = 4\right)$.

Phenotypes of animals in subsequent generation*s* were calculated as described for the base population with the exception that the true breeding values of the *i*^th^ animal, a_*i*_, was sampled as ${\displaystyle {\mathrm{a}}_i}\sim N\left(\mathrm{\frac{1}{2}}\left({\displaystyle {\mathrm{a}}_{\mathrm{s}i}}+{\displaystyle {\mathrm{a}}_{\mathrm{d}i}}\right),\sqrt{\mathrm{\frac{1}{2}}\left(1-{\displaystyle {\overline{\mathrm{F}}}_i}\right)}\right),$ where a_s*i*_ and a_d*i*_ are additive-genetic values of the sire, s_*i*_, and the dam, d_*i*_ of animal *i*, and $\overline{{\displaystyle {F}_i}}$ is the average inbreeding coefficient of s_*i*_ and d_*i*_.

### Prediction

BLUP-breeding values were predicted by fitting an animal model to the phenotypes. The model was:

$$ \mathbf{y}=\mathbf{X}\mathbf{b}+\mathbf{Z}\mathbf{a}+\mathbf{e}, $$

where **y** is a vector of phenotypes observed for selection candidates and ancestral animals, **b** is a vector of fixed birth-time effects, **a** is a vector of random animal effects, **e** is a vector of residual errors, and **X** and **Z** are incidence matrices.

The (co)variance structure used to predict the breeding values was:

$$ \left(\begin{array}{c}\hfill \mathbf{a}\hfill \\ {}\hfill \mathbf{e}\hfill \end{array}\right)\sim N\left(\left[\begin{array}{c}\hfill \mathbf{0}\hfill \\ {}\hfill \mathbf{0}\hfill \end{array}\right],\left[\begin{array}{cc}\hfill \mathbf{A}{\displaystyle {\sigma}_a^2}\hfill & \hfill \mathbf{0}\hfill \\ {}\hfill \mathbf{0}\hfill & \hfill \mathbf{I}{\displaystyle {\sigma}_e^2}\hfill \end{array}\right]\right), $$

where **A** is the additive-relationship matrix and **I** is an identity matrix. The variances, ${\displaystyle {\sigma}_a^2}$ and ${\displaystyle {\sigma}_e^2},$ were the same as those used to sample animals.

### Optimum-contribution selection

OCS allocated matings to selection candidates at time *t* by maximising the quadratic function, U_*t*_, with respect to c:

1 $$ {\displaystyle {\mathrm{U}}_t}\left(\mathbf{c}\right)=\mathbf{c}\mathbf{\hbox{'}}\widehat{\mathbf{a}}-\frac{\omega }{{\displaystyle {\mathrm{L}}^2}}\left(\mathbf{c}+\mathbf{P}\mathbf{v}\right)\mathbf{\hbox{'}}\mathbf{A}\left(\mathbf{c}+\mathbf{P}\mathbf{v}\right) $$

where **c** is a *n* vector of genetic contributions to the new cohort and the number of matings allocated to each candidate is a linear function of these contributions, **â** is a *n* vector of BLUP-breeding values, ω is the penalty applied to the average relationship of the current generation, L is the generation interval, **v** is a *k* vector of lifetime-breeding profiles or expected relative contributions to future age-classes, **P** is a *n* x *k* matrix of contributions to each age-class of animals in the current generation, **A** is a *n* x *n* matrix of additive-genetic relationships, *n* is the total number of animals in the population pedigree, which includes selection candidates, immature offspring, and all ancestors traced back from these animals, and *k* is the number of sex-age classes in the current generation. Using these definitions, it follows that **c’â** is the average breeding value of the new cohort, (**c + Pv**)/L is a vector of contributions to the current generation, **Pv**/L is a vector of contributions made to the current generation before time *t*, and ((**c** + **Pv**)’**A**(**c** + **Pv**))/L^2^ is the average relationship of the current generation. Our method of carrying out OCS is similar to that of Wray and Goddard [1], who also applied a penalty to average relationships, but it differs from that of Meuwissen [2], who constrained rates of inbreeding to pre-defined levels. In the remainder of this section, we describe the parameters, **c**, **v**, **P**, and L, and the methods that we used to impose the restrictions during optimisation.

#### Genetic contributions.

The vector of genetic contributions, **c**, was solved with linear constraints imposed. Let elements of **c**, c_m*p*_ and c_f*q*_, be the contributions of the *p*^th^ male and *q*^th^ female, then ${\displaystyle {\mathrm{c}}_{\mathrm{m}p}}=\left\{0,\frac{1}{600},\frac{2}{600},\dots, \frac{300}{600}\right\},{\displaystyle {\mathrm{c}}_{\mathrm{f}q}}=\left\{0,\frac{1}{600}\right\},{\displaystyle {\sum}_{p=1}^{{\displaystyle {n}_{\mathrm{m}}}}{\displaystyle {\mathrm{c}}_{\mathrm{m}p}}}=0.5,$ and ${\displaystyle {\sum}_{q=1}^{{\displaystyle {n}_{\mathrm{f}}}}{\displaystyle {\mathrm{c}}_{\mathrm{f}q}}}=0.5$ with c_m*p*_ = 0 and c_f*q*_ = 0 for males and females that were not candidates for selection, and *n*_m_ and *n*_f_ are the total numbers of males and females in the population pedigree (*n* = *n*_m_ + *n*_f_). The contributions allocated to the *p*^th^ male and *q*^th^ female were transformed to numbers of matings by 600 · c_m*p*_ and 600 · c_f*q*_.

#### Lifetime-breeding profiles.

The lifetime-breeding profile of animals at age *l* is the proportion of mating opportunities during their lifetime that was expected to be realised beyond age *l* [3,5]. Males, which were candidates for selection at ages 3 to 5, had *k*_m_ = 5 ages with lifetime-breeding profiles. These were at ages 0 to 4. Females were candidates at ages 4 to 6. They had *k*_f_ = 6 ages with lifetime-breeding profiles at ages 0 to 5. Older males and females did not have lifetime-breeding profiles because males and females were no longer candidates beyond ages 5 and 6.

The vector of lifetime-breeding profiles was defined as $$ \mathbf{v}=\mathrm{\frac{1}{2}}\left[\begin{array}{c}\hfill {\displaystyle {\mathbf{v}}_{\mathrm{m}}}\hfill \\ {}\hfill {\displaystyle {\mathbf{v}}_{\mathrm{f}}}\hfill \end{array}\right], $$ where **v**_m_ and **v**_f_ are *k*_m_ = 5 and *k*_f_ = 6 (*k* = *k*_m_ + *k*_f_ = 11) vectors of lifetime-breeding profiles for males at ages 0 to 4 and females at ages 0 to 5, such that,

$$ {\displaystyle {\mathbf{v}}_{\mathrm{m}}}=\left[\begin{array}{c}\hfill {\displaystyle {\mathrm{lbp}}_{\mathrm{m}0}}\hfill \\ {}\hfill {\displaystyle {\mathrm{lbp}}_{\mathrm{m}1}}\hfill \\ {}\hfill {\displaystyle {\mathrm{lbp}}_{\mathrm{m}2}}\hfill \\ {}\hfill {\displaystyle {\mathrm{lbp}}_{\mathrm{m}3}}\hfill \\ {}\hfill {\displaystyle {\mathrm{lbp}}_{\mathrm{m}4}}\hfill \end{array}\right],{\displaystyle {\mathbf{v}}_{\mathrm{f}}}=\left[\begin{array}{c}\hfill {\displaystyle {\mathrm{lbp}}_{\mathrm{f}0}}\hfill \\ {}\hfill {\displaystyle {\mathrm{lbp}}_{\mathrm{f}1}}\hfill \\ {}\hfill {\displaystyle {\mathrm{lbp}}_{\mathrm{f}2}}\hfill \\ {}\hfill {\displaystyle {\mathrm{lbp}}_{\mathrm{f}3}}\hfill \\ {}\hfill {\displaystyle {\mathrm{lbp}}_{\mathrm{f}4}}\hfill \\ {}\hfill {\displaystyle {\mathrm{lbp}}_{\mathrm{f}5}}\hfill \end{array}\right], $$and lbp_m*j*_ and lbp_f*j*_ are the lifetime-breeding profiles of males and females at age *j*. Elements lbp_m*j*_ and lbp_f*j*_ were calculated as ${\displaystyle {\mathrm{lbp}}_{mj}}=1-{\displaystyle {\sum}_{u=0}^{j-1}{\displaystyle {\upvarepsilon}_{\mathrm{m}u}}}$ and ${\displaystyle {\mathrm{lbp}}_{fj}}=1-{\displaystyle {\sum}_{u=0}^{j-1}{\displaystyle {\upvarepsilon}_{\mathrm{f}u}}},$ where ε_m*u*_ and ε_f*u*_ are the proportions of offspring from males and females at age *u*. The first three elements of **v**_m_ and the first four of **v**_f_ were 1 because males at ages 0 to 2 and females at ages 0 to 3 were immature offspring that were too young to be selection candidates. All of their mating opportunities were realised beyond ages 2 and 3.

#### Genetic contributions to animals in the current generation.

The matrix **P** was defined as $$ \mathbf{P}=\left[\begin{array}{cc}\hfill {\displaystyle {\mathbf{P}}_{\mathrm{m}}}\hfill & \hfill \mathbf{0}\hfill \\ {}\hfill \mathbf{0}\hfill & \hfill {\displaystyle {\mathbf{P}}_{\mathrm{f}}}\hfill \end{array}\right], $$ where **P**_m_ and **P**_f_ are *n*_m_ x *k*_m_ = *n*_m_ x 5 and *n*_f_ x *k*_f_ = *n*_f_ x 6 matrices. **P**_m_ contains genetic contributions of the *n*_m_ males to the *k*_m_ age-classes with lifetime-breeding profiles [3,5]. **P**_f_ contains contributions of the *n*_f_ females to the *k*_f_ age-classes. Matrices **P**_m_ and **P**_f_ have the following form:

$$ {\displaystyle {\mathbf{P}}_{\mathrm{m}}}=\left[\begin{array}{ccccc}\hfill {\displaystyle {\mathbf{0}}_{{\displaystyle {n}_{\mathrm{m}l}}}}\hfill & \hfill {\displaystyle {\mathbf{0}}_{{\displaystyle {n}_{\mathrm{m}l}}}}\hfill & \hfill {\displaystyle {\mathbf{0}}_{{\displaystyle {n}_{\mathrm{m}l}}}}\hfill & \hfill {\displaystyle {\mathbf{0}}_{{\displaystyle {n}_{\mathrm{m}l}}}}\hfill & \hfill {\displaystyle {\mathbf{0}}_{{\displaystyle {n}_{\mathrm{m}l}}}}\hfill \\ {}\hfill \vdots \hfill & \hfill \vdots \hfill & \hfill \vdots \hfill & \hfill \vdots \hfill & \hfill \vdots \hfill \\ {}\hfill {\displaystyle {\mathbf{0}}_{{\displaystyle {n}_{\mathrm{m}9}}}}\hfill & \hfill {\displaystyle {\mathbf{0}}_{{\displaystyle {n}_{\mathrm{m}9}}}}\hfill & \hfill {\displaystyle {\mathbf{0}}_{{\displaystyle {n}_{\mathrm{m}9}}}}\hfill & \hfill {\displaystyle {\mathbf{0}}_{{\displaystyle {n}_{\mathrm{m}9}}}}\hfill & \hfill {\displaystyle {\mathbf{c}}_{{\displaystyle {n}_{\mathrm{m}9}},4}}\hfill \\ {}\hfill {\displaystyle {\mathbf{0}}_{{\displaystyle {n}_{\mathrm{m}8}}}}\hfill & \hfill {\displaystyle {\mathbf{0}}_{{\displaystyle {n}_{\mathrm{m}8}}}}\hfill & \hfill {\displaystyle {\mathbf{0}}_{{\displaystyle {n}_{\mathrm{m}8}}}}\hfill & \hfill {\displaystyle {\mathbf{c}}_{{\displaystyle {n}_{\mathrm{m}8}},3}}\hfill & \hfill {\displaystyle {\mathbf{c}}_{{\displaystyle {n}_{\mathrm{m}8}},4}}\hfill \\ {}\hfill {\displaystyle {\mathbf{0}}_{{\displaystyle {n}_{\mathrm{m}7}}}}\hfill & \hfill {\displaystyle {\mathbf{0}}_{{\displaystyle {n}_{\mathrm{m}7}}}}\hfill & \hfill {\displaystyle {\mathbf{c}}_{{\displaystyle {n}_{\mathrm{m}7}},2}}\hfill & \hfill {\displaystyle {\mathbf{c}}_{{\displaystyle {n}_{\mathrm{m}7}},3}}\hfill & \hfill {\displaystyle {\mathbf{c}}_{{\displaystyle {n}_{\mathrm{m}7}},4}}\hfill \\ {}\hfill {\displaystyle {\mathbf{0}}_{{\displaystyle {n}_{\mathrm{m}6}}}}\hfill & \hfill {\displaystyle {\mathbf{c}}_{{\displaystyle {n}_{\mathrm{m}6}},1}}\hfill & \hfill {\displaystyle {\mathbf{c}}_{{\displaystyle {n}_{\mathrm{m}6}},2}}\hfill & \hfill {\displaystyle {\mathbf{c}}_{{\displaystyle {n}_{\mathrm{m}6}},3}}\hfill & \hfill {\displaystyle {\mathbf{0}}_{{\displaystyle {n}_{\mathrm{m}6}}}}\hfill \\ {}\hfill {\displaystyle {\mathbf{0}}_{{\displaystyle {n}_{\mathrm{m}5}}}}\hfill & \hfill {\displaystyle {\mathbf{c}}_{{\displaystyle {n}_{\mathrm{m}5}},1}}\hfill & \hfill {\displaystyle {\mathbf{c}}_{{\displaystyle {n}_{\mathrm{m}5}},2}}\hfill & \hfill {\displaystyle {\mathbf{0}}_{{\displaystyle {n}_{\mathrm{m}5}}}}\hfill & \hfill {\displaystyle {\mathbf{0}}_{{\displaystyle {n}_{\mathrm{m}5}}}}\hfill \\ {}\hfill {\displaystyle {\mathbf{0}}_{{\displaystyle {n}_{\mathrm{m}4}}}}\hfill & \hfill {\displaystyle {\mathbf{c}}_{{\displaystyle {n}_{\mathrm{m}4}},1}}\hfill & \hfill {\displaystyle {\mathbf{0}}_{{\displaystyle {n}_{\mathrm{m}4}}}}\hfill & \hfill {\displaystyle {\mathbf{0}}_{{\displaystyle {n}_{\mathrm{m}4}}}}\hfill & \hfill {\displaystyle {\mathbf{0}}_{{\displaystyle {n}_{\mathrm{m}4}}}}\hfill \\ {}\hfill {\displaystyle {\mathbf{0}}_{{\displaystyle {n}_{\mathrm{m}3}}}}\hfill & \hfill {\displaystyle {\mathbf{0}}_{{\displaystyle {n}_{\mathrm{m}3}}}}\hfill & \hfill {\displaystyle {\mathbf{0}}_{{\displaystyle {n}_{\mathrm{m}3}}}}\hfill & \hfill {\displaystyle {\mathbf{0}}_{{\displaystyle {n}_{\mathrm{m}3}}}}\hfill & \hfill {\displaystyle {\mathbf{0}}_{{\displaystyle {n}_{\mathrm{m}3}}}}\hfill \\ {}\hfill {\displaystyle {\mathbf{0}}_{{\displaystyle {n}_{\mathrm{m}2}}}}\hfill & \hfill {\displaystyle {\mathbf{0}}_{{\displaystyle {n}_{\mathrm{m}2}}}}\hfill & \hfill {\displaystyle {\mathbf{0}}_{{\displaystyle {n}_{\mathrm{m}2}}}}\hfill & \hfill {\displaystyle {\mathbf{0}}_{{\displaystyle {n}_{\mathrm{m}2}}}}\hfill & \hfill {\displaystyle {\mathbf{0}}_{{\displaystyle {n}_{\mathrm{m}2}}}}\hfill \\ {}\hfill {\displaystyle {\mathbf{0}}_{{\displaystyle {n}_{\mathrm{m}1}}}}\hfill & \hfill {\displaystyle {\mathbf{0}}_{{\displaystyle {n}_{\mathrm{m}1}}}}\hfill & \hfill {\displaystyle {\mathbf{0}}_{{\displaystyle {n}_{\mathrm{m}1}}}}\hfill & \hfill {\displaystyle {\mathbf{0}}_{{\displaystyle {n}_{\mathrm{m}1}}}}\hfill & \hfill {\displaystyle {\mathbf{0}}_{{\displaystyle {n}_{\mathrm{m}1}}}}\hfill \end{array}\right] $$ and

$$ {\displaystyle {\mathbf{P}}_{\mathrm{f}}}=\left[\begin{array}{cccccc}\hfill {\displaystyle {\mathbf{0}}_{{\displaystyle {n}_{\mathrm{f}l}}}}\hfill & \hfill {\displaystyle {\mathbf{0}}_{{\displaystyle {n}_{\mathrm{f}l}}}}\hfill & \hfill {\displaystyle {\mathbf{0}}_{{\displaystyle {n}_{\mathrm{f}l}}}}\hfill & \hfill {\displaystyle {\mathbf{0}}_{{\displaystyle {n}_{\mathrm{f}l}}}}\hfill & \hfill {\displaystyle {\mathbf{0}}_{{\displaystyle {n}_{\mathrm{f}l}}}}\hfill & \hfill {\displaystyle {\mathbf{0}}_{{\displaystyle {n}_{\mathrm{f}l}}}}\hfill \\ {}\hfill \vdots \hfill & \hfill \vdots \hfill & \hfill \vdots \hfill & \hfill \vdots \hfill & \hfill \vdots \hfill & \hfill \vdots \hfill \\ {}\hfill {\displaystyle {\mathbf{0}}_{{\displaystyle {n}_{\mathrm{f}11}}}}\hfill & \hfill {\displaystyle {\mathbf{0}}_{{\displaystyle {n}_{\mathrm{f}11}}}}\hfill & \hfill {\displaystyle {\mathbf{0}}_{{\displaystyle {n}_{\mathrm{f}11}}}}\hfill & \hfill {\displaystyle {\mathbf{0}}_{{\displaystyle {n}_{\mathrm{f}11}}}}\hfill & \hfill {\displaystyle {\mathbf{0}}_{{\displaystyle {n}_{\mathrm{f}11}}}}\hfill & \hfill {\displaystyle {\mathbf{c}}_{{\displaystyle {n}_{\mathrm{f}11}},5}}\hfill \\ {}\hfill {\displaystyle {\mathbf{0}}_{{\displaystyle {n}_{\mathrm{f}10}}}}\hfill & \hfill {\displaystyle {\mathbf{0}}_{{\displaystyle {n}_{\mathrm{f}10}}}}\hfill & \hfill {\displaystyle {\mathbf{0}}_{{\displaystyle {n}_{\mathrm{f}10}}}}\hfill & \hfill {\displaystyle {\mathbf{0}}_{{\displaystyle {n}_{\mathrm{f}10}}}}\hfill & \hfill {\displaystyle {\mathbf{c}}_{{\displaystyle {n}_{\mathrm{f}10}},4}}\hfill & \hfill {\displaystyle {\mathbf{c}}_{{\displaystyle {n}_{\mathrm{f}10}},5}}\hfill \\ {}\hfill {\displaystyle {\mathbf{0}}_{{\displaystyle {n}_{\mathrm{f}9}}}}\hfill & \hfill {\displaystyle {\mathbf{0}}_{{\displaystyle {n}_{\mathrm{f}9}}}}\hfill & \hfill {\displaystyle {\mathbf{0}}_{{\displaystyle {n}_{\mathrm{f}9}}}}\hfill & \hfill {\displaystyle {\mathbf{c}}_{{\displaystyle {n}_{\mathrm{f}9}},3}}\hfill & \hfill {\displaystyle {\mathbf{c}}_{{\displaystyle {n}_{\mathrm{f}9}},4}}\hfill & \hfill {\displaystyle {\mathbf{c}}_{{\displaystyle {n}_{\mathrm{f}9}},5}}\hfill \\ {}\hfill {\displaystyle {\mathbf{0}}_{{\displaystyle {n}_{\mathrm{f}8}}}}\hfill & \hfill {\displaystyle {\mathbf{0}}_{{\displaystyle {n}_{\mathrm{f}8}}}}\hfill & \hfill {\displaystyle {\mathbf{c}}_{{\displaystyle {n}_{\mathrm{f}8}},2}}\hfill & \hfill {\displaystyle {\mathbf{c}}_{{\displaystyle {n}_{\mathrm{f}8}},3}}\hfill & \hfill {\displaystyle {\mathbf{c}}_{{\displaystyle {n}_{\mathrm{f}8}},4}}\hfill & \hfill {\displaystyle {\mathbf{0}}_{{\displaystyle {n}_{\mathrm{f}8}}}}\hfill \\ {}\hfill {\displaystyle {\mathbf{0}}_{{\displaystyle {n}_{\mathrm{f}7}}}}\hfill & \hfill {\displaystyle {\mathbf{c}}_{{\displaystyle {n}_{\mathrm{f}7}},1}}\hfill & \hfill {\displaystyle {\mathbf{c}}_{{\displaystyle {n}_{\mathrm{f}7}},2}}\hfill & \hfill {\displaystyle {\mathbf{c}}_{{\displaystyle {n}_{\mathrm{f}7}},3}}\hfill & \hfill {\displaystyle {\mathbf{0}}_{{\displaystyle {n}_{\mathrm{f}7}}}}\hfill & \hfill {\displaystyle {\mathbf{0}}_{{\displaystyle {n}_{\mathrm{f}7}}}}\hfill \\ {}\hfill {\displaystyle {\mathbf{0}}_{{\displaystyle {n}_{\mathrm{f}6}}}}\hfill & \hfill {\displaystyle {\mathbf{c}}_{{\displaystyle {n}_{\mathrm{f}6}},1}}\hfill & \hfill {\displaystyle {\mathbf{c}}_{{\displaystyle {n}_{\mathrm{f}6}},2}}\hfill & \hfill {\displaystyle {\mathbf{0}}_{{\displaystyle {n}_{\mathrm{f}6}}}}\hfill & \hfill {\displaystyle {\mathbf{0}}_{{\displaystyle {n}_{\mathrm{f}6}}}}\hfill & \hfill {\displaystyle {\mathbf{0}}_{{\displaystyle {n}_{\mathrm{f}6}}}}\hfill \\ {}\hfill {\displaystyle {\mathbf{0}}_{{\displaystyle {n}_{\mathrm{f}5}}}}\hfill & \hfill {\displaystyle {\mathbf{c}}_{{\displaystyle {n}_{\mathrm{f}5}},1}}\hfill & \hfill {\displaystyle {\mathbf{0}}_{{\displaystyle {n}_{\mathrm{f}5}}}}\hfill & \hfill {\displaystyle {\mathbf{0}}_{{\displaystyle {n}_{\mathrm{f}5}}}}\hfill & \hfill {\displaystyle {\mathbf{0}}_{{\displaystyle {n}_{\mathrm{f}5}}}}\hfill & \hfill {\displaystyle {\mathbf{0}}_{{\displaystyle {n}_{\mathrm{f}5}}}}\hfill \\ {}\hfill {\displaystyle {\mathbf{0}}_{{\displaystyle {n}_{\mathrm{f}4}}}}\hfill & \hfill {\displaystyle {\mathbf{0}}_{{\displaystyle {n}_{\mathrm{f}4}}}}\hfill & \hfill {\displaystyle {\mathbf{0}}_{{\displaystyle {n}_{\mathrm{f}4}}}}\hfill & \hfill {\displaystyle {\mathbf{0}}_{{\displaystyle {n}_{\mathrm{f}4}}}}\hfill & \hfill {\displaystyle {\mathbf{0}}_{{\displaystyle {n}_{\mathrm{f}4}}}}\hfill & \hfill {\displaystyle {\mathbf{0}}_{{\displaystyle {n}_{\mathrm{f}4}}}}\hfill \\ {}\hfill {\displaystyle {\mathbf{0}}_{{\displaystyle {n}_{\mathrm{f}3}}}}\hfill & \hfill {\displaystyle {\mathbf{0}}_{{\displaystyle {n}_{\mathrm{f}3}}}}\hfill & \hfill {\displaystyle {\mathbf{0}}_{{\displaystyle {n}_{\mathrm{f}3}}}}\hfill & \hfill {\displaystyle {\mathbf{0}}_{{\displaystyle {n}_{\mathrm{f}3}}}}\hfill & \hfill {\displaystyle {\mathbf{0}}_{{\displaystyle {n}_{\mathrm{f}3}}}}\hfill & \hfill {\displaystyle {\mathbf{0}}_{{\displaystyle {n}_{\mathrm{f}3}}}}\hfill \\ {}\hfill {\displaystyle {\mathbf{0}}_{{\displaystyle {n}_{\mathrm{f}2}}}}\hfill & \hfill {\displaystyle {\mathbf{0}}_{{\displaystyle {n}_{\mathrm{f}2}}}}\hfill & \hfill {\displaystyle {\mathbf{0}}_{{\displaystyle {n}_{\mathrm{f}2}}}}\hfill & \hfill {\displaystyle {\mathbf{0}}_{{\displaystyle {n}_{\mathrm{f}2}}}}\hfill & \hfill {\displaystyle {\mathbf{0}}_{{\displaystyle {n}_{\mathrm{f}2}}}}\hfill & \hfill {\displaystyle {\mathbf{0}}_{{\displaystyle {n}_{\mathrm{f}2}}}}\hfill \\ {}\hfill {\displaystyle {\mathbf{0}}_{{\displaystyle {n}_{\mathrm{f}1}}}}\hfill & \hfill {\displaystyle {\mathbf{0}}_{{\displaystyle {n}_{\mathrm{f}1}}}}\hfill & \hfill {\displaystyle {\mathbf{0}}_{{\displaystyle {n}_{\mathrm{f}1}}}}\hfill & \hfill {\displaystyle {\mathbf{0}}_{{\displaystyle {n}_{\mathrm{f}1}}}}\hfill & \hfill {\displaystyle {\mathbf{0}}_{{\displaystyle {n}_{\mathrm{f}1}}}}\hfill & \hfill {\displaystyle {\mathbf{0}}_{{\displaystyle {n}_{\mathrm{f}1}}}}\hfill \end{array}\right]. $$

The first column of **P**_m_ and **P**_f_ represents the genetic contributions of males and females to the new cohort and is, therefore, 0. The second and subsequent columns of **P**_m_ represent the contributions of males to animals at ages 1 to 4. The second and subsequent columns of **P**_f_ represent the contributions of females to animals at ages 1 to 5. ${\displaystyle {\mathbf{0}}_{{\displaystyle {n}_{\mathrm{m}l}}}}$ and ${\displaystyle {\mathbf{0}}_{{\displaystyle {n}_{\mathrm{f}l}}}}$ are *n*_m*l*_ and *n*_f*l*_ vectors of 0s, where *n*_m*l*_ and *n*_f*l*_ are the numbers of males and females at age *l*. ${\displaystyle {\mathbf{c}}_{{\displaystyle {n}_{\mathrm{m}l}},j}}$ and ${\displaystyle {\mathbf{c}}_{{\displaystyle {n}_{\mathrm{f}l}},j}}$ are *n*_m*l*_ and *n*_f*l*_ vectors of contributions, where each element of ${\displaystyle {\mathbf{c}}_{{\displaystyle {n}_{\mathrm{m}l}},j}}$ and ${\displaystyle {\mathbf{c}}_{{\displaystyle {n}_{\mathrm{f}l}},j}}$ is the contribution of the *p*^th^ male (*p* = 1,… *n*_m*l*_) or *q*^th^ female (*q* = 1,… *n*_f*l*_) at age *l* to animals at age *j*.

#### Generation interval.

Generation interval, L, represents the average age of animals when their offspring were born (i.e., number of times for a generation to replicate itself). It was calculated as the sum of the elements of **v**, such that, $\mathrm{L}={\displaystyle {\sum}_{j=1}^k{\displaystyle {\mathbf{v}}_j}}=\frac{1}{2}\left({\displaystyle {\sum}_{j=1}^{{\displaystyle {k}_m}}{\displaystyle {\mathbf{v}}_{\mathrm{m}j}}}+{\displaystyle {\sum}_{j=1}^{{\displaystyle {k}_f}}{\displaystyle {\mathbf{v}}_{\mathrm{f}j}}}\right).$

#### Imposing restrictions on U_*t*_(c).

Restrictions were imposed during optimisation of U_*t*_(**c**) by fixing elements of **c** and **P**. Elements of **c** were fixed when imposing restrictions that removed solutions from the solution space. Elements of **P** were fixed for the restriction that removed records of selection decisions made at earlier times.

*Truncated dams* and *dams unknown*. In the breeding scheme *truncated dams*, where sires were selected by OCS and 300 dams were truncation selected, elements of **c** associated with truncation-selected dams were fixed to 1/600 (i.e., each truncation-selected dam contributed one mating). Elements of **c** were set to 0 for all other females. In the breeding scheme *dams unknown*, where the truncation-selected dams were not known before carrying out OCS of sires, all elements of **c** associated with females were fixed to 0 with the exception of an unrelated ‘dummy’ dam, which was added to the dataset with a contribution of 0.5.

*One*-*chance OCS of sires*, *pre*-*selection of sires*, and *sire multiples*. Restrictions were imposed on females by fixing elements of **c** to 1/600 for truncation-selected dams and 0 for all other females. In the breeding scheme *one*-*chance OCS of sires*, restrictions were imposed on males by fixing elements of **c** to 0 for all males that were not at age 3. In the breeding scheme *pre*-*selection of sires*, only males that were pre-selected were candidates for OCS. Elements of **c** associated with all other males were fixed to 0. In the breeding scheme *sire multiples*, elements of **c** associated with male candidates were restricted to 0, *x*/600, 2*x*/600, … 0.5, where *x* is the sire multiple (*x* = 2, 5, 10, 20, 50, and 100).

*Offspring unknown*. Contributions to immature offspring were removed by fixing columns 2 and 3 of **P**_m_ and columns 2–4 of **P**_f_ to 0.

### Data analyses

For each breeding scheme, we plotted the long-term rate of genetic gain realised at the penalty that maximised long-term rate of genetic gain for the trait under selection. Long-term rates were assessed as the proportion of additional gain realised, where additional gain was the difference in the long-term rates of genetic gain realised by *unrestricted OCS* at penalty 50 and our reference point, *truncation selection* 10. Preliminary analysis showed that the long-term rate of genetic gain realised by *unrestricted OCS* was maximised at penalty 50. Our reasoning for choosing *truncation selection* 10 as a reference point is outlined in the Appendix. The Appendix also highlights that choosing a reference point was subjective since it is difficult to find conventional breeding schemes with truncation selection that match OCS.

We also present findings that provide insight into the mechanisms that underlie OCS with restrictions:

- Penalties that maximised long-term rates of genetic gain.
- Short-term rates of genetic gain and inbreeding.
- Plot of short-term rates of genetic gain against short-term rates of inbreeding realised at the penalties that maximised long-term rates of genetic gain. We overlaid this plot with the short-term response frontier for *unrestricted OCS*, where the short-term response frontier is short-term rate of genetic gain realised at each penalty plotted as a function of short-term rate of inbreeding.
- Short-term generation intervals.
- Short-term numbers of sires with allocated matings.
- Short and long-term response frontiers for *unrestricted OCS*.

Short and long-term rates refer to animals born at times *t* = 26 to 35 (approximately generations 6 to 8) and *t* = 91 to 100 (approximately generations 23 to 25). Rates of genetic gain were calculated as the linear regression of S_*t*_ on *t*, where S_*t*_ is the average true-breeding value of animals born at time *t*. Rates of inbreeding were calculated as 1-exp(β), where β is the linear regression of ln(1-F_*t*_) on *t* and F_*t*_ is the average level of inbreeding for animals born at time *t*. The rates of genetic gain and inbreeding were scaled by setting to 100 the long-term rates of genetic gain and inbreeding realised by *unrestricted OCS* at penalty 50. Preliminary analysis showed that rate of genetic gain at 100 was equivalent to 0.215 genetic-standard deviations per time and approximately 0.9 genetic-standard deviations per generation. Rate of inbreeding at 100 was equivalent to 0.0020 per time and approximately 0.008 per generation. All results are presented as means (± s.d.) of the 100 simulation replicates.

Rates of genetic gain and inbreeding are presented as functions of time because OCS maximises rates of genetic gain in each cohort. We acknowledge the arguments for presenting rates of inbreeding per generation, namely that genetic variation erodes and mutations accumulate per generation. However, preliminary analyses showed that generation interval did not differ markedly between schemes. The relative rates of inbreeding between schemes were, therefore, similar when presented per time or per generation.

### Software

The schemes were simulated using the program, ADAM [10]. Each scheme replicate was initiated with a random seed. BLUP-breeding values were predicted using the program, DMU6 [11]. OCS was carried out using the program, EVA [12]. EVA maximised the quadratic function (Equation (1)) using an evolutionary algorithm [9,13].

## Results

### Long-term rates of genetic gain

**Figure 1:**
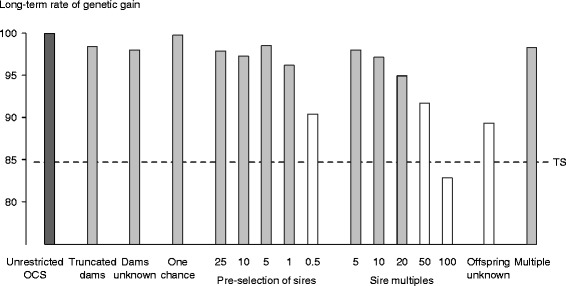
**Maximum long**-**term rates of genetic gain realised by optimum**-**contribution selection** **(OCS)** **without and with restrictions.** Breeding scheme applying OCS without restrictions is *Unrestricted OCS* (dark-shaded bar). Schemes applying OCS with restrictions are *Truncated dams*, *Dams unknown*, One-chance (representing breeding scheme, *one*-*chance OCS of sires*), *Pre*-*selection of sires* 25, 10, 5, 1, and 0.5%, *Sire multiples* 5, 10, 20, 50, and 100, *Offspring unknown*, and Multiple (*multiple restrictions*). These schemes are presented as light-shaded bars, except those that failed to realise most of the long-term genetic gain realised by *Unrestricted OCS* (unshaded bars). Long-term rate of genetic gain realised by a conventional scheme with truncation selection (TS) is presented as a reference point (dotted line). Long-term refers to generations 23 to 25 (approximately). The rates are means of 100 simulation replicates. The means had standard deviations ranging from 5.8 to 12.0. The rates were scaled by setting to 100 the maximum long-term rate of genetic gain realised by OCS without restrictions. Rate of genetic gain at 100 is equivalent to 0.215 genetic-standard deviations per time and approximately 0.9 genetic-standard deviations per generation.

OCS with restrictions realised most of the additional long-term genetic gain that was realised by OCS without restrictions (Figure 1). OCS without restrictions realised 18% additional gain, where additional gain was the difference in the long-term rates of genetic gain realised by *unrestricted OCS* at penalty 50 and our reference point, *truncation selection* 10. In all but a few exceptions, OCS with restrictions at penalties that maximised long-term rates of genetic gain realised 67 to 99% of this additional gain. Even *multiple restrictions*, where several of the restrictions that remove solutions from the solution space were imposed simultaneously, realised 89% of the additional gain. The exceptions were *pre*-*selection of sires* 0.5%, *sire multiples* 50 and 100, and *offspring unknown*. Imposing these restrictions realised only −12 to 46% of the additional gain.

The following sections present findings that provide insight into the mechanisms that underlie OCS with restrictions.

### Penalties that maximised long-term rates of genetic gain

The penalties that maximised long-term rates of genetic gain in each breeding scheme with OCS were 50 and 100 (Table 2). The exceptions were *pre*-*selection of sires* 0.5 and 1.0% and *multiple restrictions*. In *pre*-*selection of sires* 0.5 and 1.0%, the penalties that maximised long-term rates of genetic gain were 5000 and 200. It was 200 in *multiple restrictions*.

**Table 2:** **Output parameters that provide insight into the mechanisms underlying optimum**-**contribution selection** (**OCS**) **with restrictions**

**Breeding scheme**	**Penalty**	∆**G**_**short**_	∆**F**_**short**_	**L**_**short**_	**nSires**_**short**_
*Unrestricted OCS*	50	109 ± 9.7	114 ± 27.0	4.1 ± 0.04	13.3 ± 1.19
*Truncated dams*	50	109 ± 9.6	132 ± 45.7	4.1 ± 0.04	12.8 ± 1.10
*Dams unknown*	100	102 ± 7.6	87 ± 30.2	4.2 ± 0.04	23.4 ± 1.72
*One chance OCS of sires*	50	106 ± 8.7	95 ± 22.2	3.8 ± 0.01	17.6 ± 1.25
*Pre*-*selection of sires* 25%	50	108 ± 10.2	137 ± 51.0	4.1 ± 0.04	12.7 ± 1.08
*Pre*-*selection of sires* 10%	100	103 ± 8.0	88 ± 26.9	4.1 ± 0.03	20.0 ± 2.03
*Pre*-*selection of sires* 5%	100	107 ± 10.5	105 ± 31.1	4.0 ± 0.03	17.9 ± 2.07
*Pre*-*selection of sires* 1%	200	111 ± 10.7	192 ± 60.8	3.9 ± 0.03	11.7 ± 1.69
*Pre*-*selection of sires* 0.5%	5000	110 ± 10.6	268 ± 85.2	3.9 ± 0.04	7.3 ± 0.70
*Sire multiples* 5	50	108 ± 10.2	139 ± 50.6	4.1 ± 0.05	11.9 ± 1.04
*Sire multiples* 10	50	108 ± 9.7	137 ± 43.3	4.1 ± 0.05	10.8 ± 0.77
*Sire multiples* 20	50	107 ± 12.1	134 ± 52.0	4.1 ± 0.05	8.9 ± 0.58
*Sire multiples* 50	50	104 ± 11.1	148 ± 51.4	4.0 ± 0.06	5.5 ± 0.21
*Sire multiples* 100	50	95 ± 12.2	172 ± 55.9	4.0 ± 0.07	3.0 ± 0.00
*Offspring unknown*	50	106 ± 15.1	189 ± 76.1	4.3 ± 0.05	9.7 ± 1.11
*Multiple restrictions*	50	108 ± 8.5	102 ± 24.4	3.8 ± 0.03	13.6 ± 0.80
*Truncation selection 10*		112 ± 10.0	413 ± 161.1	4.1 ± 0.03	10

Breeding scheme applying OCS without restrictions is *Unrestricted OCS*. All other schemes applied OCS with restrictions, except *Truncation selection 10*, which is a conventional scheme with truncation selection. The output parameters are penalty on average relationship that maximised long-term rate of genetic gain, short-term rates of genetic gain and inbreeding (∆G_short_, ∆F_short_), short-term generation interval (L_short_), and short-term number of sires with allocated matings per selection time (nSires_short_), where short and long-term refer to generations 6 to 8 and generations 23 to 25 (approximately). The rates of genetic gain and inbreeding, generation intervals, and numbers of sires are presented as means ± s.d. of 100 simulation replicates. Rates of genetic gain and inbreeding were scaled by setting to 100 the long-term rates of genetic gain and inbreeding realised by *Unrestricted OCS* at penalty 50. Rate of genetic gain at 100 is equivalent to 0.215 genetic-standard deviations per time and approximately 0.9 genetic-standard deviations per generation. Rate of inbreeding at 100 is equivalent to 0.0020 per time and approximately 0.008 per generation on the observed scale.

### Short-term rates of genetic gain and inbreeding

**Figure 2:**
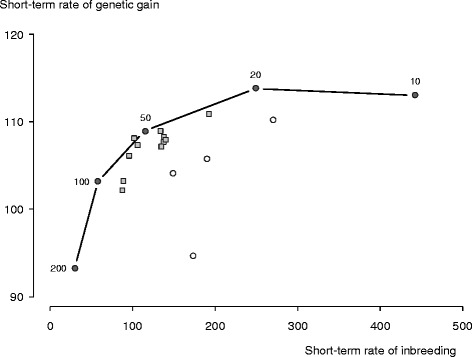
**Short**-**term rates of genetic gain and inbreeding realised by optimum**-**contribution selection** **(OCS)** **with restrictions.** Short-term rates realised by breeding schemes applying OCS with restrictions are at penalties on average relationship that maximised long-term rates of genetic gain, where short and long-term refer to generations 6 to 8 and generations 23 to 25 (approximately). The rates are represented by shaded squares, except for schemes that failed to realise most of the long-term genetic gain realised by OCS without restrictions, namely *pre*-*selection of sires* 0.5%, *sire multiples* 50 and 100, and *offspring unknown* (empty circles). The plot is overlaid with the short-term response frontier for OCS without restrictions (line with filled circles). The short-term response frontier is short-term rate of genetic gain realised at five penalties plotted as a function of short-term rate of inbreeding. The penalties are 10, 20, 50, 100, and 200. The long-term rate of genetic gain realised by OCS without restrictions was maximised at penalty 50. The rates are means of 100 simulation replicates. The rates were scaled by setting to 100 the long-term rates of genetic gain and inbreeding realised by OCS without restrictions at penalty 50. Rate of genetic gain at 100 is equivalent to 0.215 genetic-standard deviations per time and approximately 0.9 genetic-standard deviations per generation. Rate of inbreeding at 100 is equivalent to 0.0020 per time and approximately 0.008 per generation on the observed scale.

OCS with restrictions realised short-term rates of genetic gain and inbreeding that differed from the short-term rates realised by OCS without restrictions (Table 2). At the penalties that maximised long-term genetic gain, OCS with restrictions realised 94 to 102% of the short-term rates of genetic gain and 76 to 235% of the short-term rates of inbreeding realised by *unrestricted OCS*. Not only did these rates differ, they formed a distinct pattern when we plotted the short-term rates of genetic gain against short-term rates of inbreeding for each restriction and overlaid this plot with the short-term response frontier for *unrestricted OCS* (Figure 2). In this plot, the short-term rates of genetic gain and inbreeding realised by OCS with most of our restrictions aligned themselves along the response frontier and were centred round the short-term rates realised by *unrestricted OCS* at penalty 50, the breeding scheme that maximised long-term rates of genetic gain. The exceptions were *pre*-*selection of sires* 0.5%, *sire multiples* 50 and 100, and *offspring unknown*, namely the same restrictions that failed to realise most of the long-term genetic gain. The short-term rates realised by OCS with these restrictions deviated furthest from the response frontier and the short-term rates realised by *unrestricted OCS* at penalty 50.

### Short-term generation intervals

Short-term generation intervals at the penalties that maximised long-term rates of genetic gain ranged from 4.0 to 4.1 times (Table 2). The exceptions were *one*-*chance OCS of sires*, *pre*-*selection of sires* 0.5 and 1.0%, which had slightly shorter generation intervals (3.8 to 3.9). *Dams unknown* and *offspring unknown* had slightly longer generation intervals (4.2 and 4.3).

### Short-term numbers of sires with allocated matings

Matings were allocated to approximately 13 sires during the short-term time period of *unrestricted OCS* at penalty 50 (Table 2). Similar numbers of sires were allocated matings in *truncated dams*, *pre*-*selection of sires* 25%, *sire multiples* 5, and *multiple restrictions* at the penalties that maximised long-term genetic gain (11.9 to 13.6 sires per time). More sires were allocated matings in *dams unknown* and *one*-*chance OCS of sires* (23.4 and 17.6). Fewer were allocated matings in *offspring unknown* (9.7). In *pre*-*selection of sires*, the number of sires allocated matings increased from 12.7 to 20.0 as the proportion of pre-selected sires fell from 25% to 10%. It then decreased to 7.3 as the proportion fell to 0.5%. The number of allocated matings in *sire multiples* decreased from 11.9 to 3.0 as the multiple was increased from 5 to 100.

### Short and long-term response frontiers

**Figure 3:**
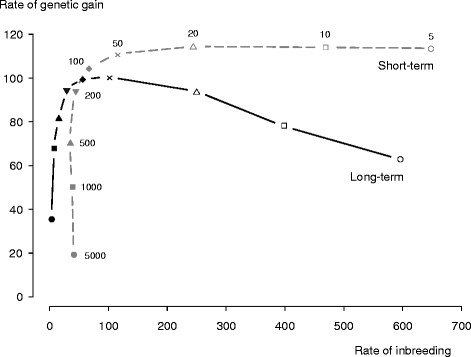
**Short and long**-**term response frontiers realised by optimum**-**contribution selection** **(OCS)** **without restrictions.** The short and long-term response frontiers are short and long-term rates of genetic gain realised at nine penalties on average relationship plotted as a function of short and long-term rates of inbreeding, where short and long-term refer to generations 6 to 8 and generations 23 to 25 (approx.). The penalties are 5 (○), 10 (□), 20 (∆), 50 (Χ), 100 (♦), 200 (▼), 500 (▲), 1000 (■), and 5000 (●). The rates were scaled by setting to 100 the long-term rates of genetic gain and inbreeding realised at penalty 50. Rate of genetic gain at 100 is equivalent to 0.215 genetic-standard deviations per time and approximately 0.9 genetic-standard deviations per generation. Rate of inbreeding at 100 is equivalent to 0.0020 per time and approximately 0.008 per generation on the observed scale.

Long-term rates of genetic gain were maximised at penalties that promoted short-term rates of genetic gain without substantially eroding additive-genetic variation. This is illustrated by our short and long-term response frontiers for *unrestricted OCS* (Figure 3). In unrestricted OCS, where long-term rate of genetic gain was maximised at penalty 50, a decrease in penalty from 50 to 5 reduced long-term rate of genetic gain to 62%. This was because a decrease in penalty from 50 to 5 realised no more than 105% of the short-term genetic gain realised at penalty 50, but it increased the short-term rate of inbreeding by 564%. On the other hand, increasing the penalty from 50 to 5000 reduced long-term genetic gain to only 34%. This was because an increase in penalty from 50 to 5000 realised only 16% of the short-term genetic gain realised at penalty 50, although it decreased short-term rate of inbreeding to 8%.

There were two further observations from the frontiers. First, short and long-term rates of inbreeding were more sensitive to changes in penalty than short and long-term rates of genetic gain at penalties smaller than 100. At penalties larger than 100, rates of genetic gain were more sensitive than rates of inbreeding. Second, short-term rates of genetic gain were higher than long-term rates at penalties smaller than 200. For penalties larger than 200, long-term rates of genetic gain were higher than short-term rates.

## Discussion

Our findings supported our hypothesis that OCS with restrictions imposed during optimisation realises most of the long-term genetic gain realised by OCS without restrictions. Realising 67 to 99% of the additional gain with many of our restrictions demonstrates that OCS is a robust selection method. This robustness was even evident with *multiple restrictions*, where several restrictions that remove solutions from the solution space were imposed simultaneously. In breeding schemes for which OCS cannot be applied optimally because of biological and logistical restrictions, OCS with restrictions provides a useful alternative. Not only does it realise most of the long-term genetic gain, OCS with restrictions enables OCS to be tailored to individual breeding schemes, where the optimum number of matings, given the restrictions, can be readily allocated to available selection candidates. It was only when the restrictions became too strict, whereby all solutions near the optimum solution were removed from the solution space or records of selection decisions made at earlier selection times were removed, that we failed to realise most of the additional gain. So, provided the restrictions are not too strict, most of the long-term genetic gain realised by OCS without restrictions can be realised by OCS with restrictions imposed during optimisation.

OCS with many of our restrictions realised most of the long-term genetic gain for two reasons. First, solutions near the optimum still existed in the solution spaces after the restrictions had been imposed. By applying different penalties to the average relationship, we gave OCS the opportunity to search the reduced solution spaces to find alternate solutions. Second, OCS was able to correct for earlier selection decisions by taking into account that some animals had already contributed to the current generation. When we applied different penalties to find alternate solutions, we also shifted the balance between the short-term rates of genetic gain and inbreeding. This was illustrated by our plot of the short-term rates of genetic gain against the short-term rates of inbreeding realised by each restriction. These rates laid along the short-term response frontier for OCS without restrictions and centred round the rates realised by the optimum solution. This implies that, to cope with restrictions, we need to give OCS the opportunity to shift the balance between short-term rates of genetic gain and inbreeding. It also underlines that short-term rates of genetic gain and inbreeding are merely intermediary parameters, a ‘means to an end’, given that long-term rate of genetic gain is the goal of most breeding schemes. Therefore, OCS with restrictions should always realise most of the long-term genetic gain provided solutions near the optimum still exist in the solution space, OCS is able to correct for selection decisions made at earlier selection times, and OCS is given the opportunity to shift the balance between short-term rates of genetic gain and inbreeding in search of alternate solutions.

It was for these reasons that OCS was able to cope with the first of our restrictions, *truncated dams* and *dams unknown*, where restrictions were imposed on female candidates. *Truncated dams* removed potential solutions from the solution space by fixing elements of **c** in Equation (1) that were associated with truncation-selected dams. Elements for all other females were set to 0. In *dams unknown*, all elements of **c** associated with females were fixed to 0. There were two striking features of these restrictions. First, OCS only needed to be applied to males to realise most of the long-term genetic gain realised by OCS without restrictions. This was because the intensity of selection and the variation in genetic contributions were higher for sires than dams. Selection of sires had a greater impact on rates of genetic gain and inbreeding. Second, the identity of the truncation-selected dams was not needed by OCS to realise most of the genetic gain. The impact of the selected sires on the genetic merit of the new cohort did not depend on which dams were truncation-selected, while there was sufficient information from earlier selection decisions for OCS to estimate the average relationship of the current generation via **P** in Equation (1). **P** contained genetic contributions to the current generation, including contributions from the parents of the truncation-selected dams and dams that had already generated offspring. Thus, OCS can cope with restrictions imposed on female candidates in breeding schemes where the selection intensity for males is higher than for females.

The capacity for OCS to cope with restrictions was further evident when restrictions were also imposed on male candidates. Solutions near the optimum still existed when elements of **c** associated with both males and females were fixed by *one*-*chance OCS of sires*, *pre*-*selection of sires* 25, 10, 5, and 1%, and *sire multiples* 5, 10, and 20. With *one*-*chance OCS of sires*, only young males were allocated matings. Elements of **c** associated with older males were fixed to 0. OCS coped because young males tended to have higher breeding values than older males. There was also enough genetic diversity to constrain average relationship despite the fact that fewer males were available for selection, young males were from fewer families, and the available males tended to be related. Solutions still existed with *pre*-*selection of sires* 25, 10, 5, and 1% despite the fact that only males that were pre-selected based on breeding value were allocated matings by OCS. Elements of **c** associated with all other males were fixed to 0. OCS did this by overcoming several challenges posed by pre-selection: fewer available males for OCS, increased between-family variation for male candidates, and increased average relationship of the male candidates. Pre-selected males also tended to be related to the truncation-selected dams, because truncation-selected dams also ranked highest for breeding value. *Sire multiples* 5, 10, and 20 realised most of the long-term gain although increases in the multiple reduced the maximum number of sires that could have been allocated matings. All males were candidates for OCS, but as the multiple increased, fewer sires and, in turn, fewer ancestors could have made genetic contributions to each generation. So, not only can OCS cope with restrictions imposed on female candidates, it can also handle restrictions imposed on males.

Although OCS coped with many of our restrictions, there was a limit where the restrictions became too strict and we failed to realise most of the long-term genetic gain. *Pre*-*selection of sires* 0.5% and *sire multiples* 50 and 100 failed to realise most of the gain because they removed all solutions near the optimum from the solution space. *Offspring unknown* removed records of selection decisions made at earlier times. These restrictions impacted on short-term rates of genetic gain, but their greatest impact was through increased rates of inbreeding, which eroded additive-genetic variation. *Pre*-*selection of sires* 0.5% increased rates of inbreeding because only about 11 males were available for OCS and these males tended to be related. No allocation of matings to these males could have realised low rates of inbreeding. This was supported by the fact that the penalty that maximised long-term genetic gain for *pre*-*selection of sires* 0.5% was 5000, the largest penalty that we applied to the average relationship. In *sire multiples* 50 and 100, OCS could only allocate matings to a maximum of six and three sires. With so few sires and ancestors making genetic contributions to each generation, an accumulation of inbreeding was unavoidable. In *offspring unknown*, contributions to young, immature offspring were removed by fixing columns of **P** to 0. This led OCS to underestimate the average relationship of the current generation. OCS was more likely to allocate matings to sires that had already contributed to the current generation, which increased rates of inbreeding. These explanations were reinforced by our plot of short-term rates of genetic gain against short-term rates of inbreeding. The rates realised by OCS with *pre*-*selection of sires* 0.5%, *sire multiples* 50 and 100, and *offspring unknown* deviated from the short-term response frontier for OCS without restrictions and from the rates realised by the optimum solution. Thus, although OCS is a robust selection system, it does have limits. It is sensitive to restrictions that remove all solutions near the optimum from the solution space or remove records of earlier selection decisions.

Long-term rates of genetic gain were maximised at penalties that struck an appropriate balance between short-term rates of genetic gain and inbreeding. This was illustrated by our short and long-term response frontiers for *unrestricted OCS*. The main feature of these frontiers was that the penalty that maximised long-term rates of genetic gain realised almost as much short-term genetic gain as OCS at smaller penalties. Sacrificing small amounts of short-term gain led to vastly reduced rates of inbreeding. This highlights that OCS with the appropriate penalty promotes short-term genetic gain while maintaining additive-genetic variation and the potential to realise long-term gain. Therefore, it makes good sense to use OCS with penalties that strike an appropriate balance between short-term rates of genetic gain and inbreeding.

Not only does it make good sense to use penalties that strike an appropriate balance, the penalties should also realise acceptable rates of inbreeding. Acceptable rates can be difficult to define, given that the nature of genetic variation, the generation of new variation through mutation, and the impacts of inbreeding on fitness are poorly understood. Current knowledge suggests that rates between 0.005 and 0.01 per generation are acceptable [14]. This equates to rates between 60 and 125 per time (approximately) after we scaled our rates of inbreeding by setting to 100 the long-term rate of inbreeding realised by *unrestricted OCS* at penalty 50. Accepting Bijma’s [14] rates has two implications. First, it provides animal breeders with an additional criterion by which to define penalties for OCS. Second, Bijma’s [14] rates indicate that our findings are robust to the time horizon that we assumed for our simulations and that they generalise beyond this horizon. So, striking an appropriate balance between short-term rates of genetic gain and inbreeding, and realising rates of inbreeding that fall within acceptable levels, provide two worthwhile criteria by which to define penalties for OCS.

A further feature of the response frontiers was that small penalties resulted in short-term rates of genetic gain that were higher than long-term rates, while large penalties resulted in long-term rates that were higher than short-term rates. There are two interrelated explanations for this. First, at each penalty, variation in breeding values and variation in additive-genetic relationships decreased at different rates over time, where breeding values and additive-genetic relationships are represented by **â** and **A** in Equation (1). Second, the relative constraint of each penalty on average relationship changed as the ratio of the two variations changed over time. Decreasing the variation in breeding values faster than the variation in additive-genetic relationships increased the relative constraint on average relationship, while decreasing the variation in additive-genetic relationships faster than the variation in breeding values reduced the constraint. These two explanations mean that, at small penalties, where emphasis was on genetic gain, OCS decreased the variation in breeding values faster over time than it decreased the variation in additive-genetic relationships. Not only did this limit the potential to realise genetic gain, it increased the relative constraint on average relationship. At high penalties, where the emphasis was on constraining average relationship, variation in additive-genetic relationships decreased faster than variation in breeding values. This retained the potential to realise genetic gain while reducing the relative constraint on average relationship. This reasoning leads us to speculate that, in practice, penalties that maximise rates of long-term genetic gain are those that maintain a constant relative constraint on average relationship over time. Achieving this will involve dynamic penalties, derived at each selection time as functions of the variations in breeding values and additive-genetic relationships, given that we do not know the impact of individual penalties on long-term rates of genetic gain. Therefore, the choice of penalty plays an important role in managing variation in breeding values and additive-genetic relationships. It may even be possible to increase long-term genetic gain by applying dynamic penalties over time.

Although the restrictions that we imposed were inspired by pig breeding, OCS should also cope with these and other restrictions in most breeding schemes for two reasons. First, the principle that underlies OCS is the same for all breeding schemes, irrespective of the species, number or type of traits, phenotyping strategy, and genetic and phenotypic (co)variances. This is because OCS uses summary statistics as input. Genetic merit is an aggregate breeding value, while average relationship is derived from a matrix of genetic relationships. Second, the mechanisms by which OCS coped with each restriction apply across schemes. While the mechanisms are the same, what is sure to differ between schemes is the point at which the restrictions become too strict. For example, OCS could become sensitive to *dams unknown* in schemes where the selection intensity and variation in genetic contributions of females are increased. OCS will probably also become sensitive to *one*-*chance OCS of sires* when the interval between selection times is shortened and there are fewer male candidates available for selection at each time. Likewise, the level of pre-selection where *pre*-*selection of sires* becomes too strict will presumably vary between schemes that differ for population size, family structure, and selection intensity. Thus, the underlying mechanisms by which OCS copes with restrictions should apply across breeding schemes, but the point at which the restrictions become too strict will probably differ.

## Figures and Tables

**Figure 4:**
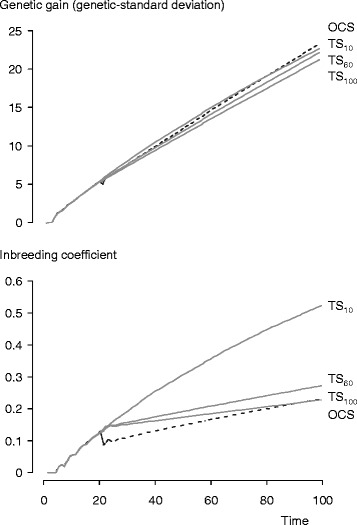
**Genetic gain and inbreeding realised by optimum-** **contribution** **(OCS)** **without restrictions and truncation selection over time.** Breeding scheme applying OCS without restrictions is represented by OCS (dotted line). Three schemes with truncation selection are *truncation selection* 10, *truncation selection* 60, and *truncation selection* 100 (TS_10_, TS_60_, TS_100_, solid lines).

## Appendix

### Choosing a reference point for OCS

Choosing a conventional breeding scheme with truncation selection as a reference point for the long-term rates of genetic gain realised by OCS was subjective. The reason was that rates and levels of genetic gain and inbreeding realised by OCS and truncation selection differed over time. It was, therefore, difficult to find a conventional breeding scheme with truncation selection that matched OCS.

This is illustrated by a preliminary analysis, where we traced the genetic gain and inbreeding realised by (i) a breeding scheme with *unrestricted OCS* at penalty 50, and (ii) three schemes with truncation selection. The three schemes with truncation selection were *truncation selection* 10, *truncation selection* 60, and *truncation selection* 100. *Unrestricted OCS* at penalty 50 and *truncation selection* 10 were simulated as described in the Methods section. *Truncation selection* 60 and *truncation selection* 100 were simulated as described for *truncation selection* 10 with the exception that 60 and 100 sires were truncation selected at each time. Genetic gain was presented on the genotypic scale and inbreeding on the observed scale.

Tracing genetic gain and inbreeding over time illustrated six points (Figure 4):

- Rate of genetic gain was not constant over time. The rate of genetic gain realised by *unrestricted OCS* fell from 0.238 genetic-standard deviations per time during times 26 to 35 to 0.215 genetic-standard deviations per time during times 91 to 100. The rate realised by *truncation selection* 10 fell from 0.252 to 0.182. It fell from 0.221 to 0.201 for *truncation selection* 60 and from 0.207 to 0.196 for *truncation selection* 100.
- *Unrestricted OCS* realised higher rates of genetic gain than truncation selection. The rate of genetic gain realised by *unrestricted OCS* during times 26 to 35 was 7.4 and 15.0% higher than *truncation selection* 60 and *truncation selection* 100 (0.238 *vs* 0.221 and 0.207 genetic-standard deviations per time). It was 7.0 and 9.7% higher than *truncation selection* 60 and *truncation selection* 100 during times 91 to 100 (0.215 *vs*. 0.201 and 0.196). The exception was *truncation selection* 10, where *unrestricted OCS* only realised higher rates of genetic gain after time 45 (approximately). The rate of genetic gain realised by *unrestricted OCS* was 5.6% lower than *truncation selection* 10 during times 26 to 35 (0.238 *vs*. 0.252). It was 18.1% higher during times 91 to 100 (0.215 *vs*. 0.181).
- *Unrestricted OCS* realised more genetic gain than truncation selection. The level of genetic gain realised by *unrestricted OCS* at time 35 was 0.4 and 4.0% higher than *truncation selection* 60 and *truncation selection* 100 (8.7 *vs*. 8.7 and 8.4 genotypic standard deviations). It was 5.0 and 9.5% higher than *truncation selection* 60 and *truncation selection* 100 at time 100 (23.3 *vs*. 22.2 and 21.3). The exception was *truncation selection* 10, where *unrestricted OCS* only realised more genetic gain after time 80. The level of genetic gain realised by *unrestricted OCS* was 5.8% lower than *truncation selection* 10 at time 35 (8.7 *vs*. 9.2). It was 2.6% higher at time 100 (23.3 *vs*. 22.7).
- Rate of inbreeding realised by *unrestricted OCS* was not constant over time. It was approximately constant for truncation selection. The rate of inbreeding realised by *unrestricted OCS* fell from 0.0023 per time during times 26 to 35 to 0.0020 per time during times 91 to 100. The rate realised by *truncation selection* 10 was approximately 0.0075 per time during times 21 to 100. It was approximately 0.0020 and 0.0014 during times 21 to 100 for *truncation selection* 60 and *truncation selection* 100.
- *Unrestricted OCS* realised lower rates of inbreeding than *truncation selection* 10, similar rates as *truncation selection* 60, and higher rates than *truncation selection* 100. The rate of inbreeding realised by *unrestricted OCS* was 70.8 and 73.4% lower than *truncation selection* 10 during times 26 to 35 and 91 to 100 (0.0023 *vs*. 0.0078 and 0.0020 *vs*. 0.0075 per time). *Unrestricted OCS* realised 11.8% more inbreeding than *truncation selection* 60 during times 26 to 35 (0.0023 *vs*. 0.0020) and 3.9% less during times 91 to 100 (0.0020 *vs*. 0.0021). It realised 75.4 and 44.2% more inbreeding than *truncation selection* 100 during times 26 to 35 and 91 to 100 (0.0023 *vs*. 0.0013 and 0.0020 *vs*. 0.0014).
- *Unrestricted OCS* realised lower levels of inbreeding than truncation selection. The level of inbreeding realised by *unrestricted OCS* at time 35 was 46.2, 27.1, and 22.4% lower than *truncation selection* 10, *truncation selection* 60, and *truncation selection* 100 (0.12 *vs*. 0.22, 0.17, and 0.16). It was 55.6 and 14.8% lower than *truncation selection* 10 and *truncation selection* 60 at time 100 (0.23 *vs*. 0.52 and 0.27) and approximately the same as *truncation selection* 100 at time 100 (0.23 *vs*. 0.23).

These points highlight that OCS and truncation selection are contrasting selection methods that realise different rates and levels of genetic gain and inbreeding. With no obvious match, we chose *truncation selection* 10 as the reference point for our simulations. Our reasoning was that, when we compared *truncation selection* 10 with *unrestricted OCS*, we saw the original motivation for OCS: sacrificing small amounts of short-term genetic gain can vastly reduce rate of inbreeding [1,2]. This promotes genetic gain while maintaining additive-genetic variation and the potential to continue realising gain. The other schemes with truncation selection also have merit as reference points. For example, *truncation selection* 60 realised similar rates of inbreeding as *unrestricted OCS*, although it did so at higher levels of inbreeding. Using *truncation selection* 60 and *truncation selection* 100 as reference points would not have changed the conclusion of our study.
